# Interactions between DC-SIGN and the envelope protein from Dengue and Zika viruses: a structural perspective based on molecular dynamics and MM/GBSA analyses

**DOI:** 10.1186/s12985-023-02251-4

**Published:** 2023-12-04

**Authors:** Bruno Stein Barbosa Menechino, Rodrigo Bentes Kato, Helena Cristina Ferreira Franz, Pedro Eduardo Almeida da Silva, Marcus Corat, Daniel Ferreira de Lima Neto

**Affiliations:** 1 Multidisciplinary Center for Biological Research - Laboratory for the Development of Biological Models, University of Campinas, 5 de Junho St., 230, Cidade Universitária, Campinas, SP 13083-877 Brazil; 2 General-Coordination of Public Health Laboratories, Department of Strategic Articulation in Health and Ambient, Ministry of Health, Brasília, Brazil

**Keywords:** DC-SIGN, Dengue, Zika, Molecular dynamics, MM/GBSA

## Abstract

Zika virus (ZIKV) and dengue virus (DENV) share a lot of similarities being both phylogenetically closely related, share the same insect vector passage for reaching the host, affinity for the same carbohydrate receptor domains (CRDs), indicating feasible competition between them on the natural field. Here, we prospected interactions of both envelope proteins with a DC-SIGN, a transmembrane c-type lectine receptor with the most implicated CRD with the Flavivirus infection presents on dendritic cells involved in viruses replication processes into the host, and among rares CRD receptors susceptible to interacting with a broad of subtypes of DENV. Protein–protein docking procedures produced structures for molecular dynamics experiments, suggesting the most energetically favorable complex. The difference found in the deltaG results prompted the experimentation with molecular dynamics. To investigate further specific residues involved with such interactions we produced a decomposition analysis using molecular dynamics of the docked proteins evaluated afterward with the Generalized Born Surface Area method. Solvent-accessible surface area (SASA) analysis for both showed very similar but with a slight reduction for ZIKV_E, which agreed with residues SASA analysis highlighting regions more exposed in the ZIVK protein than in DENV. Despite residues PHE313 is reponsible for most of the interactions with the envelope of these arboviruses, ZIKV interacted with this residue in DC-SIGN with lower energies and using more interactions with not expexted residues GLU241 and ARG386. Taken together these results suggest better competitive interaction of ZIKV with the DC-SIGN receptor, particularly in the CRD portion.

**Supplementary Information:**

The online version contains supplementary material available at 10.1186/s12985-023-02251-4.

Zika virus (ZIKV) and Dengue Virus (DENV) share a lot of similarities. Both are flaviviruses that belong to the genus Flavivirus and family Flaviviridae, are considered neglected tropical diseases and have been having a growing incidence in the last decade, and the majority of cases of these viruses are asymptomatic or mild and self managed, wich make the number of cases to be under-reported or misdiagnosed as other febrile illnesses or even other flaviviruses as it is estimated that the reported burden of dengue cases is only a small proportion of the total volume of apparent infections [1–3].

Studying the interaction of flaviviruses with white cell receptors is important for several reasons. First, understanding how viruses attach to and enter cells is essential for developing effective treatments and vaccines. Investigating specific receptors used by flaviviruses to infect white blood cells, one can develop drugs that prevent the virus from binding to the receptors, thus blocking the virus from entering the cell. Furthermore, the interaction between flaviviruses and white blood cells have important implications for the body's immune response. When white blood cells are infected by a virus, they produce inflammatory cytokines, which help fight the infection. However, in some flavivirus infections, excessive production of cytokines can lead to an overactive immune response, which can cause tissue damage in the body. Finally, studying the interaction of flaviviruses with white cell receptors help to elucidate the mechanisms underlying the pathogenesis of these viruses and provide insights into how they evolved to infect cells of the immune system. This could lead to a better understanding of how viruses spread and adapt to the host, which is important for the development of effective strategies to prevent and control flaviviral diseases [4–6].

Here we describe the interaction of DENV and ZIKV envelope proteins in a structural comparison involving homology modeling of these proteins, guided docking based on deposited crystallography, molecular dynamics of the docked complexes and for the decomposition of interacting residues we employed an analysis of the surface area accessible by the Generalized Born method.

The accession number of the sequences used in this work are QUJ10646.1(ZIKV), QEV86479.1(DENV), and NP_066978.1(DC-SIGN). First, the ZIKV envelope protein nucleotide sequence was translated to aminoacids for the input of FASTA format into SWISS-MODEL [7] (swiss] model.expansy.org) and the generation of possible models based on the data. The outputed PDB file was then uploaded to Chimera software [8] for energy minimization using 1000 steps for both steepest descent and conjugate gradient, while the other parameters remained the default. With the minimization finished, the resulting PDB file was submitted to Molprobity [9] for validation and steric evaluation where the hidrogen atoms inserted by Chimera were removed due to Molprobity’s standardization handling of electron-cloud values. With the hydrogens added the “Analyze all-atom contacts and geometry” was run with all universal and protein checkboxes selected. The PDB file extracted from Molprobity was then submited for a quality analysis using the VERIFY3D [10] and PROCHECK [11] tools from SAVES v6.0 in addition to the ProSA analysis through ProSA-Web Protein Structure plataform [12].

The docking process was repeated for both ZIKV envelope protein and DENV envelope protein against DC-SIGN. Based on the crystallography deposited under the Protein Data Bank (PDB) code 2B6B we extracted the interacting residues via LigPlot2 + [13] and used them as attractors in the guided docking made in the HADDOCK2 website [14]. The most energetically favorable conformation was used for further analyses.

The docked complexes were then prepared for simulation in the GROMACS 2021.3 package [15], all experiments were conducted for 100 ns and were prepared according to the following methodology. The docked systems were prepared in the conversion to the GROMACS format with the pdb2gmx module using the TIP3P model for the waters in the system and the chosen force field AMBER99SB-ILDN protein. A rhombic dodecahedron was selected to surround the system with a distance of 1 angstrom from the edges of the system. The system solvation was performed with the SPC226 model. A twin range cut-off of 1.0 nm was used for both Coulombic (short-range electrostatic) and Lennard–Jones nonbonded interactions, with long-range electrostatics calculated using the particle mesh Ewald (PME) algorithm. The net electric charge of the system was neutralized with sodium ions and sodium chloride salt was added to a concentration of 150 mmol. The systems were energy minimized by using the steepest descent algorithm until the maximum force converged under 10 kJ/mol. The equations of motion were integrated by using the leapfrog algorithm with a step size of 2 fs. The lengths of all bonds involving hydrogen atoms were constrained to their equilibrium values by using the LINCS algorithm. Initial velocities were obtained from a Maxwellian distribution at the temperature of 300 K, and a temperature equilibration under constant volume (NVT ensemble) trajectory was computed for 200 ps. The system was then equilibrated under constant pressure (NPT ensemble) to 1 bar employing the Berendsen barostat with a 2 ps coupling time in a trajectory computed for for 200 ps. The production molecular dynamics simulations were then carried out for 100 ns employing the Parrinello-Rahman barostat with a 2 ps coupling time to isotropically regulate pressure. The MM-GBSA approach is a powerful approach to evaluate the binding free energy in a protein–protein (PPi) system. This study adopted the GBSA method considering the time consuming PBSA alternative for PPi ensembles. The gmx_MMPBSA tool [16] was used for high-yield MM/GBSA calculations, allowing one to calculate individual energy terms and the total interaction energy, performing energy decompositions for each residue through Python scripts. All simulations were run in a 24 processor machine yelding approximately 50 ns/day production runs.

The ZIKV envelope is an outer layer of proteins that surrounds the genetic material of the virus. An important interaction occurs between the ZIKV envelope and a protein present on the surface of immune cells called DC-SIGN (Dendritic Cell-Specific Intercellular adhesion molecule-3-Grabbing Non-integrin). DC-SIGN is a cell adhesion molecule present in dendritic cells, macrophages and endothelial cells. This protein has the ability to bind to a wide variety of pathogens, including viruses such as ZIKV. The interaction between the ZIKV envelope and DC-SIGN is important because it allows the virus to bind to dendritic cells, which are one of the main types of immune cells in the human body. Binding of ZIKV to dendritic cells via DC-SIGN may allow the virus to enter the interior of dendritic cells, where it can replicate and spread. Furthermore, studies have shown that the interaction of ZIKV with DC-SIGN can also lead to an inadequate immune response, which may contribute to the pathogenesis of the disease. Binding of ZIKV to DC-SIGN can suppress the host's adaptive immune response, allowing the virus to replicate and spread more easily. A review of the putative receptors used by DENV to enter cells is reproduced in Additional file 10: Table S1.

**Table 1:** Results for the evaluations made at the PRODIGY server

Protein–protein complex	Î”G (kcal mol^-1)	Kd (M) at â„ƒ	ICs charged-charged	ICs charged-polar	ICs charged-apolar	ICs polar-polar	ICs polar-apolar	ICs apolar-apolar	NIS charged	NIS apolar
DENV_E-DC-SIGN	− 12.6	5.80E-10	10	18	25	7	24	40	25.29	39.46
ZIKV_E-DC-SIGN	− 15.2	6.60E-12	10	11	37	6	30	35	23.92	41.05

As with the ZIKV, the DENV envelope also interacts with the DC-SIGN protein present on the surface of immune cells, such as dendritic cells. This interaction occurs through hydrogen bonds formed between the virus envelope and the DC-SIGN protein. Hydrogen bonds are chemical interactions that occur between molecules that have functional groups capable of donating and receiving hydrogen ions. In the case of interaction between the DENV envelope and DC-SIGN, hydrogen bonds occur between the amino acid residues present on the surface of the viral envelope and the sugar residues present in the DC-SIGN protein. The interacting residues derived from the crystal are described in Additional file 11: Table S2.

After performing the dockings and evaluating the interaction energies on the PRODIGY server (Table 1), we performed the molecular dynamics of the complexes according to the methodology described above. We performed a production of 100 ns for each complex and the trajectories were analyzed according to the described procedures. We also compared the electrostatic maps of each envelope protein to illustrate the subtle differences between the two cases (Fig. 1).

**Fig. 1:**
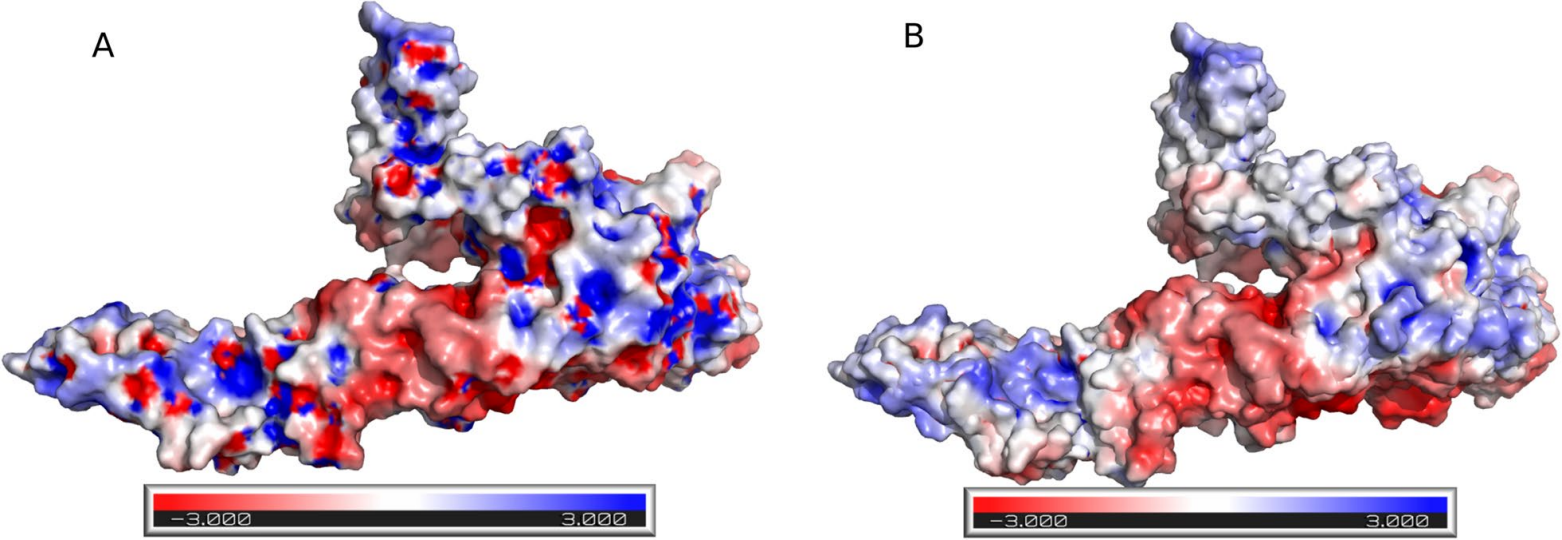
Electrostatic maps of the envelope protein of (**A**) DENV and (**B**) ZIKV. The maps are coloured according to the electrostatic potential as measured by the APBS server, red being negative and blue being positive

The regions of interaction with the DC-SIGN receptor, as observed in the crystallography used as a guide for performing the dockings, were highlighted in Additional file 1: Fig. S1 (with 3D representations in SF 2) to illustrate the comparison of the two envelopes. Note that the region of interest for the discussion is predominantly electropositive in the DENV envelope, but when comparing the same region in the ZIKV envelope, we can observe that there is a decrease in charges, not to the point of making the region electronegative as a whole, but enough for the surroundings of the interaction region to become neutral with electronegative trends (Fig. 1A–B).

The energy minimization and the subsequent NVT and NPT equilibrium steps were successfully conducted and the trajectories were produced until reaching 100 ns for the docked complexes. As shown in Fig. 2A, the solvent-accessible surface area (SASA) of both envelope proteins are very similar, and that of ZIKV showed a slight reduction in SASA after the equilibrium and burn-in period of the simulation. On the other hand, when we analyzed the SASA by residue (Fig. 2B) we noticed that some regions are more exposed in the ZIVK protein than in DENV, namely in the regions of domain I and II, responsible for the interaction with DC-SIGN as previously demonstrated for DENV. Both simulations stabilized quickly with small variations in Root Mean Square Deviation (RMSD—Fig. 2C) and interestingly we observed differences in alpha-carbon fluctuations after the simulation was completed. The ED-1 and ED-2 region of the ZIKV envelope showed greater flexibility in the range of residues from 70 to 100 whereas the following regions showed less flexibility compared to the DENV envelope (Fig. 2D). Clustering of the trajectories showed that there are 3D species at small RMSD variations in the DENV envelope, but classified as distinct, whereas the structure of the ZIKV envelope showed fluctuations but fewer 3D species. The principal components analysis of the trajectory section of the last 10 ns (RMSD stabilization) showed little variation in PC1 and PC2, as did the PCA analysis for ZIKV, however ZIKV stabilized the structure in the last 10 ns. We also performed the RMSD and RMSF, PCA and hierachical clustering analyzes in the MDTraj and Bio3D packages [17, 18], these results are described in the supplementary material (Additional files 3, 4, 5, 6, 7, 8, 9: Figs. S3, S4, S5, S6, S7, S8 and S9).

**Fig. 2:**
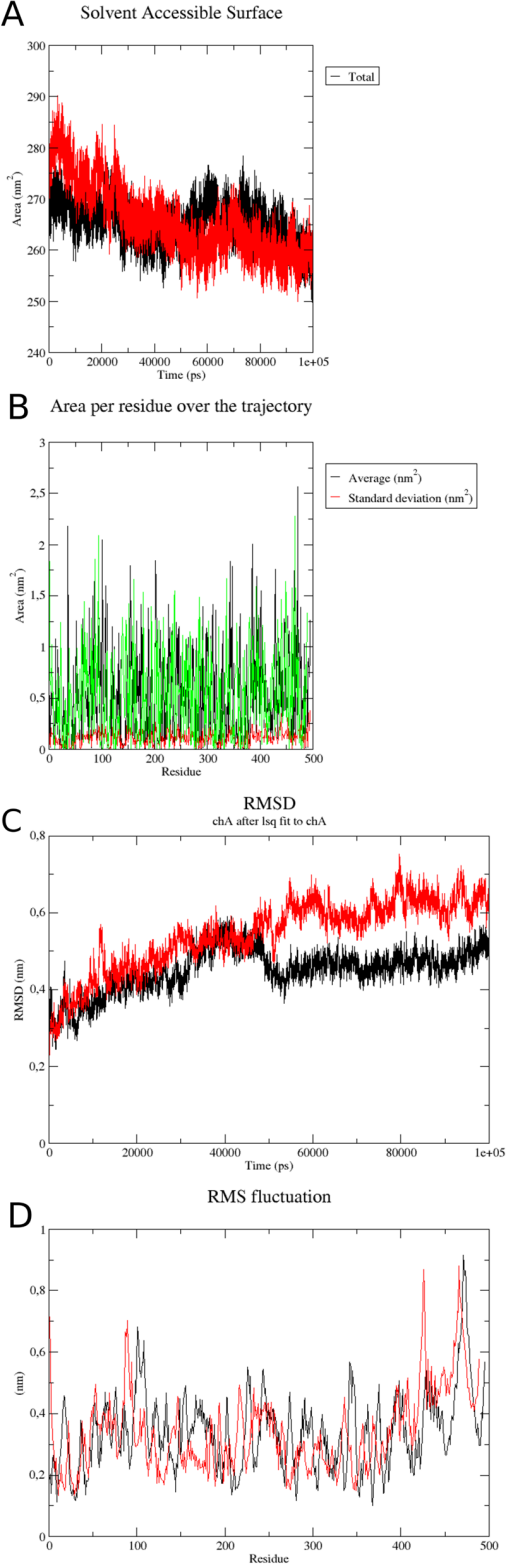
Molecular dynamics results of the docked protein–protein complex. Each run comprised 100 ns of production run and the results are based on the selection of the envelope protein for each virus, the results were then combined in GRACE graph visualization tool. **A**: Solvent acessible surface area (SASA), black for DENV and red for ZIKV, **B**: Solvent acessible surface area per residue, black for DENV and green for ZIKV, root mean square deviation for the carbon alfa backbone of DENV (black) and ZIKV (red), **C**: root mean square deviation for chain A (virus). DENV (black), ZIKV (red). **D**: root mean square fluctuation per residue for DENV (black) and ZIKV (red)

It was possible to observe that the difference in the interaction energies suggested by the PRODIGY server (Table 1) of the most energetically favorable results were also maintained during the molecular mechanics simulation, however some marked differences were also observed. Figure 3 shows these results in detail. We observed in the interaction residue decomposition that DENV strongly interacted with DC-SIGN on residues PHE96, PRO217, and TRP465 whereas ZIKV interacted better with residues ARG80, LEU82, PRO208, and ALA227. In comparison with the literature findings, the DC-SIGN residue PHE313 is responsible for most of the interactions with the envelope of these arboviruses, which was also observed here for DENV (PHE313 strongly interacted in this case with PHE96 and LYS110, with energies around − 7.5 kcal/mol) whereas ZIKV interacted with this residue in DC-SIGN at the amino acids LEU442, PHE443, GLY444, and MET446, but with lower energies than DENV). On the other hand ZIKV mounted more interactions with residues GLU241 and ARG386 with energies around − 9 kcal/mol.

**Fig. 3:**
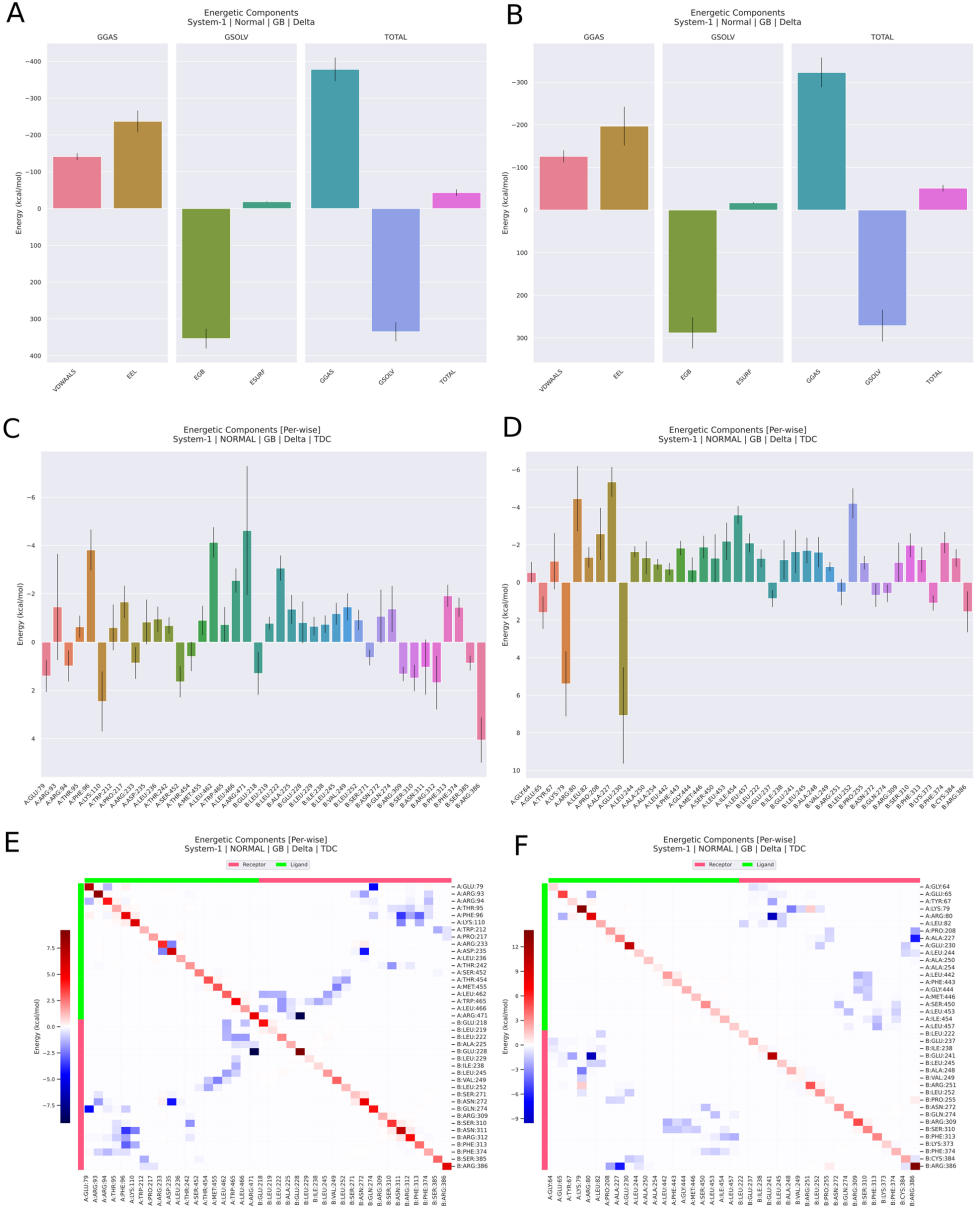
Molecular mechanics analyzed via Generalized Born Surface Area (MM/GBSA) results. **A**, **C** and **E** represents DENV results and **B**, **D**, **F** represents ZIKV results. **A**, **B**: Total decomposition analyses. **C**, **D**: Per residue decomposition analyses. **E**, **F**: Heatmap of the interaction forces per residue on the interaction interface

Taken together these results suggest better interaction of ZIKV with the DC-SIGN receptor, particularly in the CRD portion. This carbohydrate recognition motif is present on a series of receptors that these arboviruses use to internalize into host white blood cells. In view of the vast literature on the internalization of DENV mediated by DC-SIGN in dendritic cells and the resulting dispersion of this infection in the immune system, we suggest, through experiments in docking, molecular dynamics and analysis of surface accessible to the solvent by the generalized Born method that ZIKV also makes use of this receptor. This was already implied by previous works that verified the homology of the envelope proteins of these arboviruses, however the quantification of these interactions was still a gap in the literature.

Our study evaluates the interaction between the viral receptor (envelope) of DENV and ZIKV with the dendritic cell receptor DC-SIGN and for this purpose, we used homology modeling of structures derived from Brazilian sequences. A limiting point of the work was the execution of the dynamics (two trajectories for each case) without the use of molecular sampling tools such as the use of replca exchange to insert variability in the simulation. To overcome this limitation, two runs were carried out for each case. Furthermore, the work essentially focuses on the interaction with the DC-SIGN receptor, not addressing the many other receptors already documented for DENV envelope, this is due to the fact that the CRD domain is shared with other cellular receptors, making the work a little more comprehensive in this sense. Alternatively, simulations with only the CRD docked against the viral envelopes could have been approached in order to overcome the situation exposed, however, by resorting to this alternative we would lose important structural information in the result that the other residues generate in the three-dimensional configuration of the CRD itself.

This article consistently contrasts the interaction profiles of DENV and ZIKV with DC-SIGN, highlighting the unique and shared interaction patterns of these viruses. This comparison provides readers with a nuanced understanding of how two closely related viruses can exhibit differences in their energy interaction profiles. Additionally, the authors take the approach of being a detailed, data-driven exploration of the molecular interactions between proteins. Our paper also tries to contextualize its findings within the broader scientific literature bringing together evolutionary aspects of the closely related viruses used herein and structural virology, which is currently a topic of great interest.

### Supplementary Information

**Additional file 1**. **Supplementary figure 1**: ligplot representation of the hydrophobic and hydrogen bond interactions. A: DENV-DC-SIGN interactions. B: ZIKV-DC-SIGN interactions.

**Additional file 2. Supplementary figure 2**: pymol 3D representation of the hydrophobic and hydrogen bond interactions. A: DENV-DC-SIGN interactions. B: ZIKV-DC-SIGN interactions.

**Additional file 3**. **Supplementary figure 3**: DENV RMSD Average linkage hierarchical clustering produced by the MDTraj package.

**Additional file 4**. **Supplementary figure 4**: DENV Cartesian coordinate Principal Component Analysis produced by the MDTraj package.

**Additional file 5**. **Supplementary figure 5**: ZIKV RMSD Average linkage hierarchical clustering produced by the MDTraj package.

**Additional file 6**. **Supplementary figure 6**: ZIKV Cartesian coordinate Principal Component Analysis produced by the MDTraj package.

**Additional file 7**. **Supplementary figure 7**: Bio3D analyses. **A**: DENV RMSD histogram over 100 ns of molecular dynamics simulation. **B**: ZIKV RMSD histogram over 100 ns of molecular dynamics simulation. **C**: DENV RMSD over 100 ns of molecular dynamics simulation. **D**: RMSD ZIKV over 100 ns of molecular dynamics simulation. **E**: RMSF DENV over 100 ns of molecular dynamics simulation. **F**: RMSF ZIKV over 100 ns of molecular dynamics simulation.

**Additional file 8**. **Supplementary figure 8**: Bio3D analyses 2. **A**: DENV PCA (PC1, PC2 and PC3 correlations) and Eigenvalues for the 100 ns of molecular dynamics simulation. **B**: ZIKV PCA (PC1, PC2 and PC3 correlations) and Eigenvalues for the 100 ns of molecular dynamics simulation. **C**: DENV Hierarchical clustering (PC1, PC2 and PC3correlations) and Eigenvalues for the 100 ns of molecular dynamics simulation. **D**: ZIKV Hierarchical clustering (PC1, PC2 and PC3 correlations) and Eigenvalues for the 100 ns of molecular dynamics simulation. **E**: DENV -PC1 applied for residue position. **F**: ZIKV - PC1 applied for residue position.

**Additional file 9**. **Supplementary figure 9**: Residue cross correlation. **A**: DENV. **B**: ZIKV.

**Additional file 10**. **Supplementary table 1**: Putative receptors for DENV in mammalian cells.

**Additional file 11**. **Supplementary table 2**: List of interfacing residues found at ligplot+.

## Data Availability

Due to the size of the trajectory files these are available upon request to the corresponding author.
