# Supplementary figures and images for: Interactions between DC-SIGN and the envelope protein from Dengue and Zika viruses: a structural perspective based on molecular dynamics and MM/GBSA analyses
Source: Virol J. 2023 Dec 4;20:286. doi: 10.1186/s12985-023-02251-4 (PMC10696828; doi:10.1186/s12985-023-02251-4)

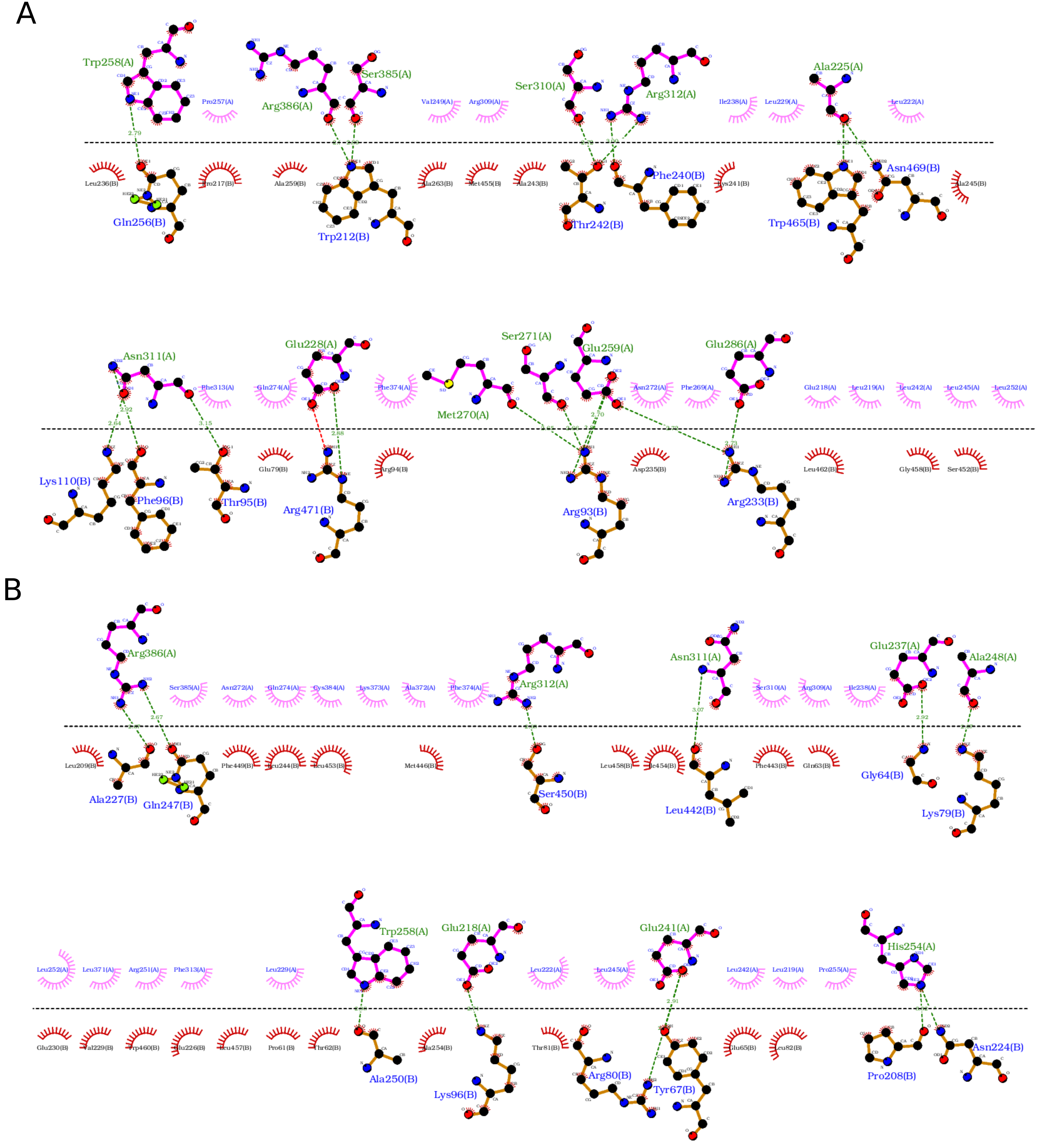

Supplement: Supplementary file 1 — Additional file 1. Supplementary figure 1: ligplot representation of the hydrophobic and hydrogen bond interactions. A: DENV-DC-SIGN interactions. B: ZIKV-DC-SIGN interactions. [file 12985_2023_2251_MOESM1_ESM.jpeg]

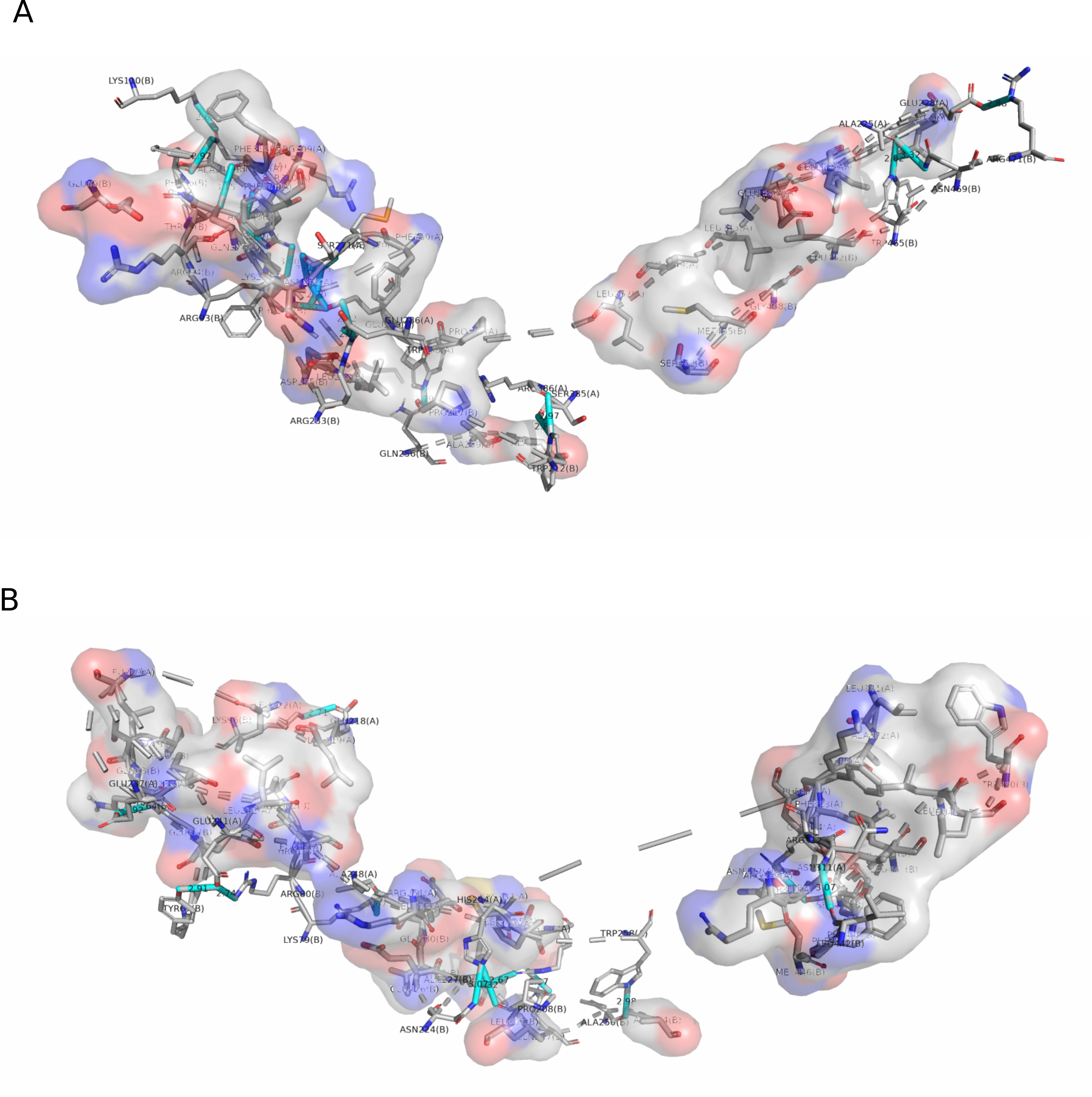

Supplement: Supplementary file 2 — Additional file 2. Supplementary figure 2: pymol 3D representation of the hydrophobic and hydrogen bond interactions. A: DENV-DC-SIGN interactions. B: ZIKV-DC-SIGN interactions. [file 12985_2023_2251_MOESM2_ESM.jpeg]

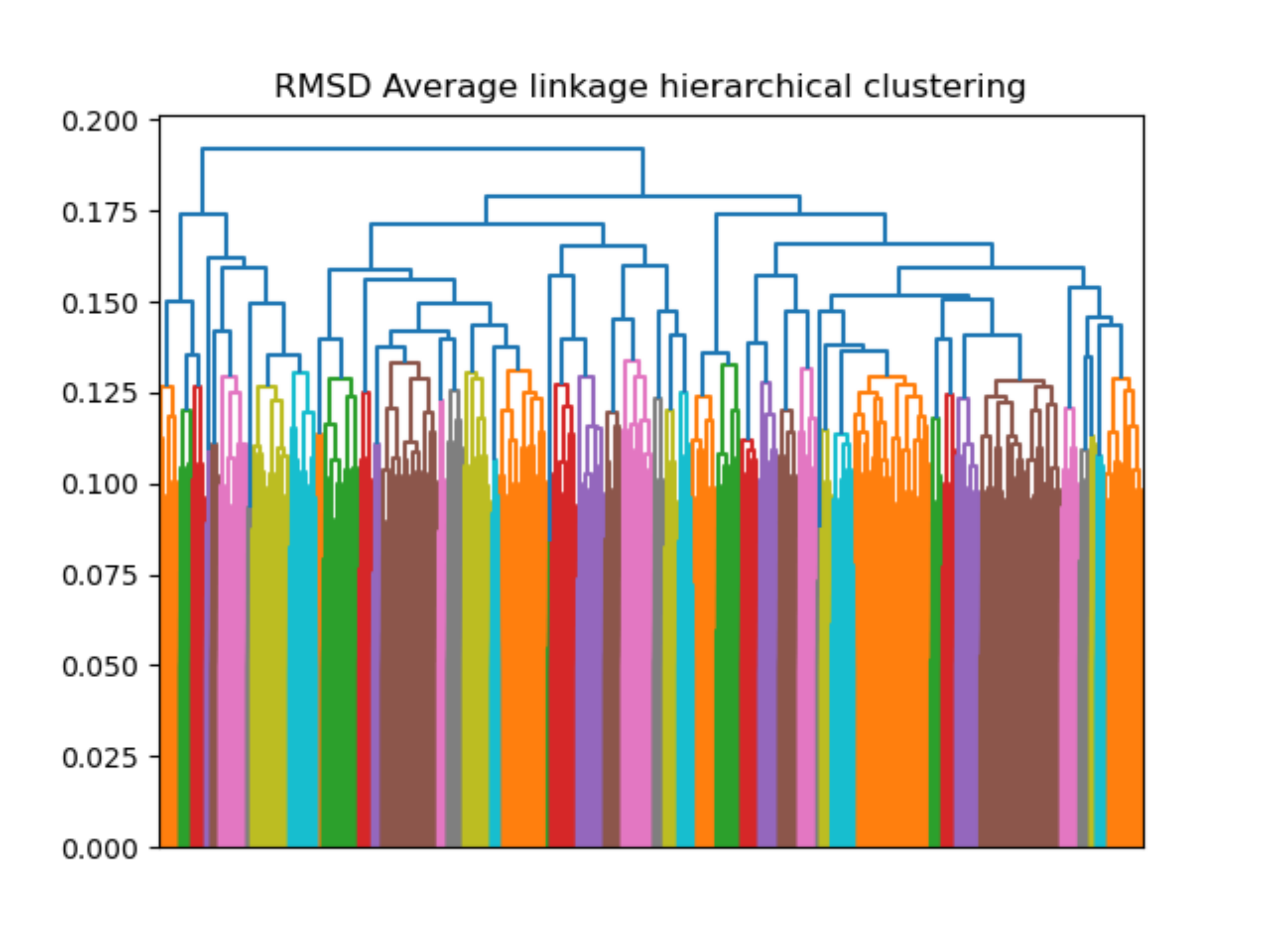

Supplement: Supplementary file 3 — Additional file 3. Supplementary figure 3: DENV RMSD Average linkage hierarchical clustering produced by the MDTraj package. [file 12985_2023_2251_MOESM3_ESM.jpeg]

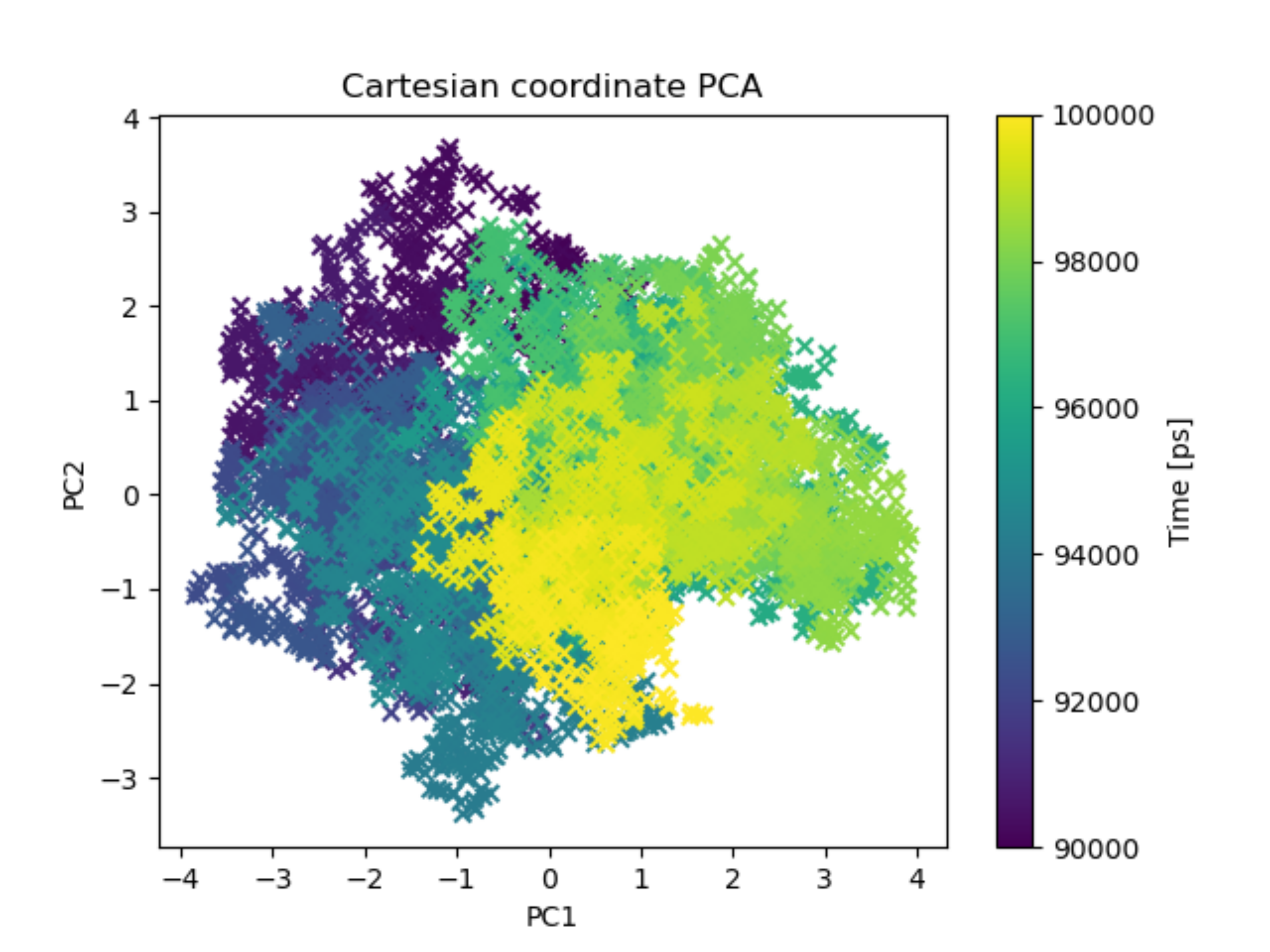

Supplement: Supplementary file 4 — Additional file 4. Supplementary figure 4: DENV Cartesian coordinate Principal Component Analysis produced by the MDTraj package. [file 12985_2023_2251_MOESM4_ESM.jpeg]

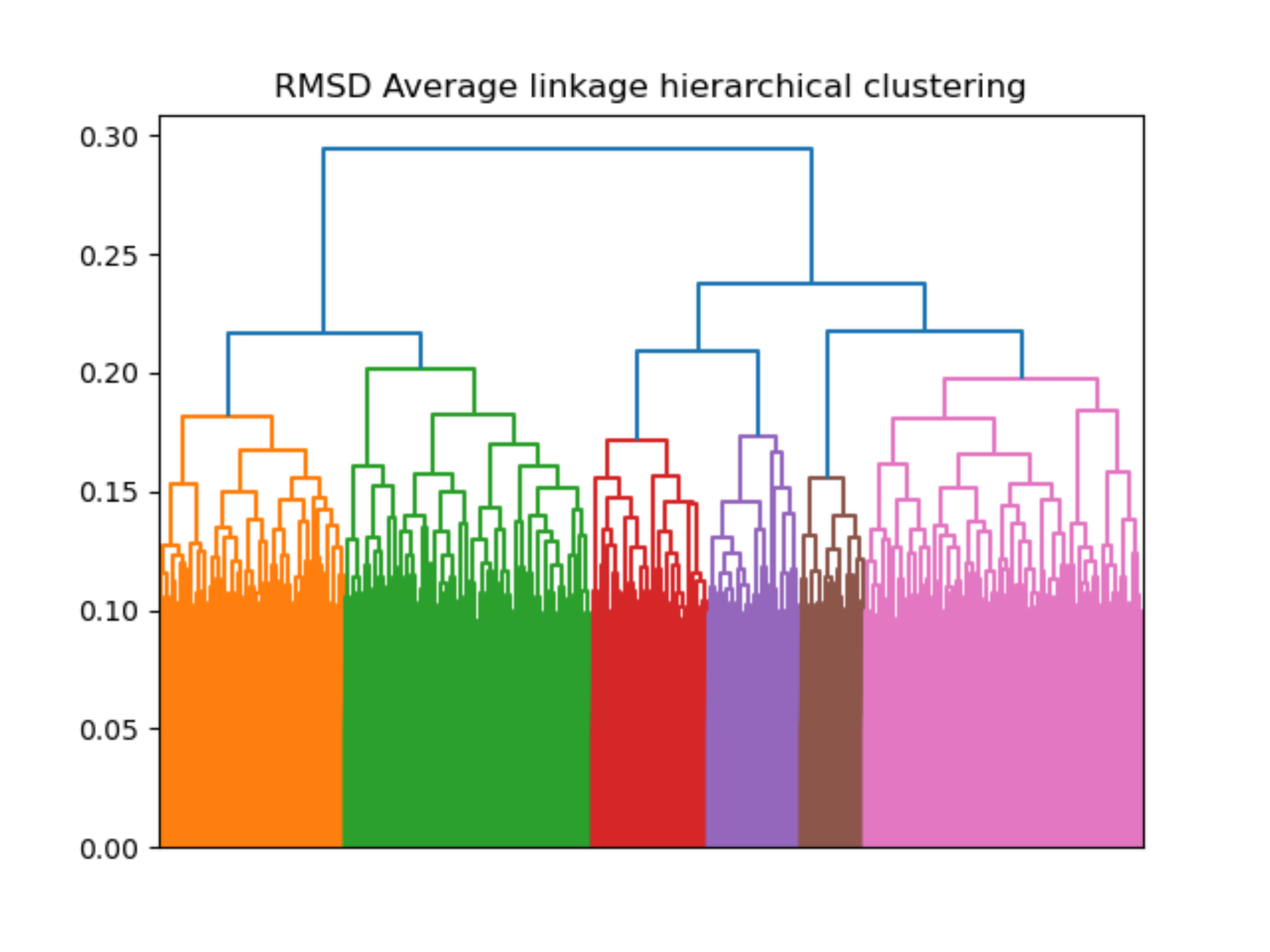

Supplement: Supplementary file 5 — Additional file 5. Supplementary figure 5: ZIKV RMSD Average linkage hierarchical clustering produced by the MDTraj package. [file 12985_2023_2251_MOESM5_ESM.jpeg]

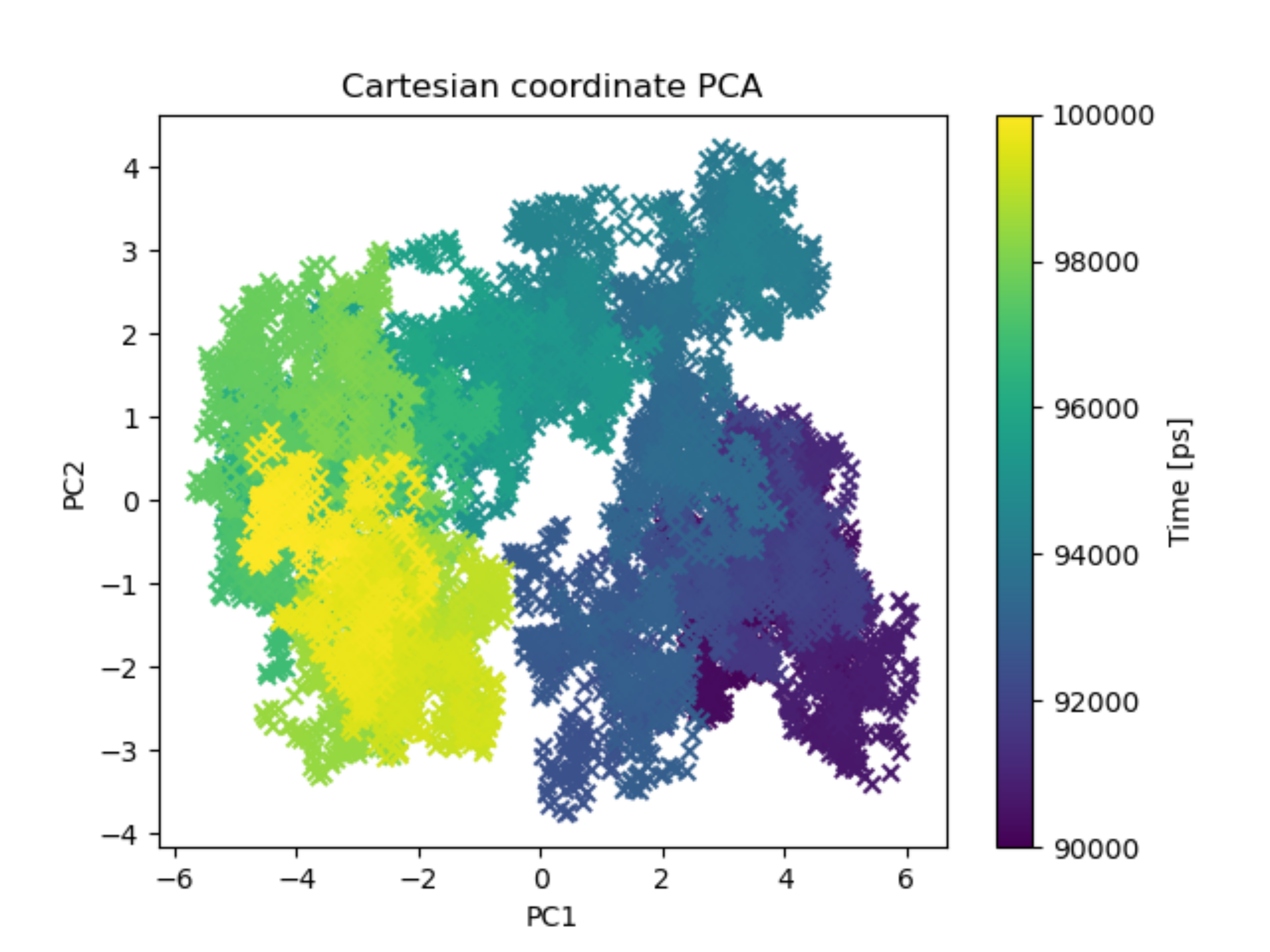

Supplement: Supplementary file 6 — Additional file 6. Supplementary figure 6: ZIKV Cartesian coordinate Principal Component Analysis produced by the MDTraj package. [file 12985_2023_2251_MOESM6_ESM.jpeg]

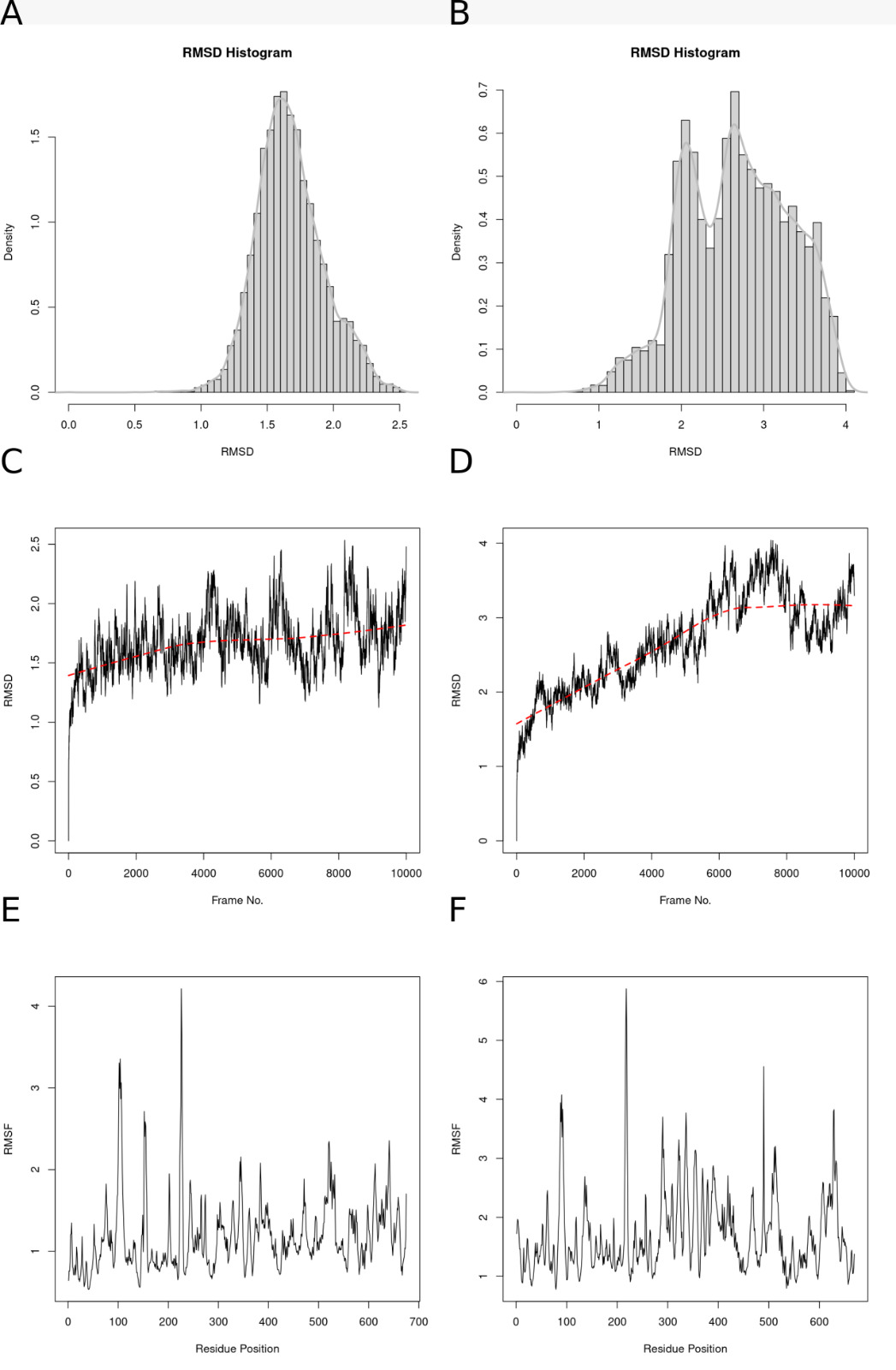

Supplement: Supplementary file 7 — Additional file 7. Supplementary figure 7: Bio3D analyses. A: DENV RMSD histogram over 100 ns of molecular dynamics simulation. B: ZIKV RMSD histogram over 100 ns of molecular dynamics simulation. C: DENV RMSD over 100 ns of molecular dynamics simulation. D: RMSD ZIKV over 100 ns of molecular dynamics simulation. E: RMSF DENV over 100 ns of molecular dynamics simulation. F: RMSF ZIKV over 100 ns of molecular dynamics simulation. [file 12985_2023_2251_MOESM7_ESM.jpeg]

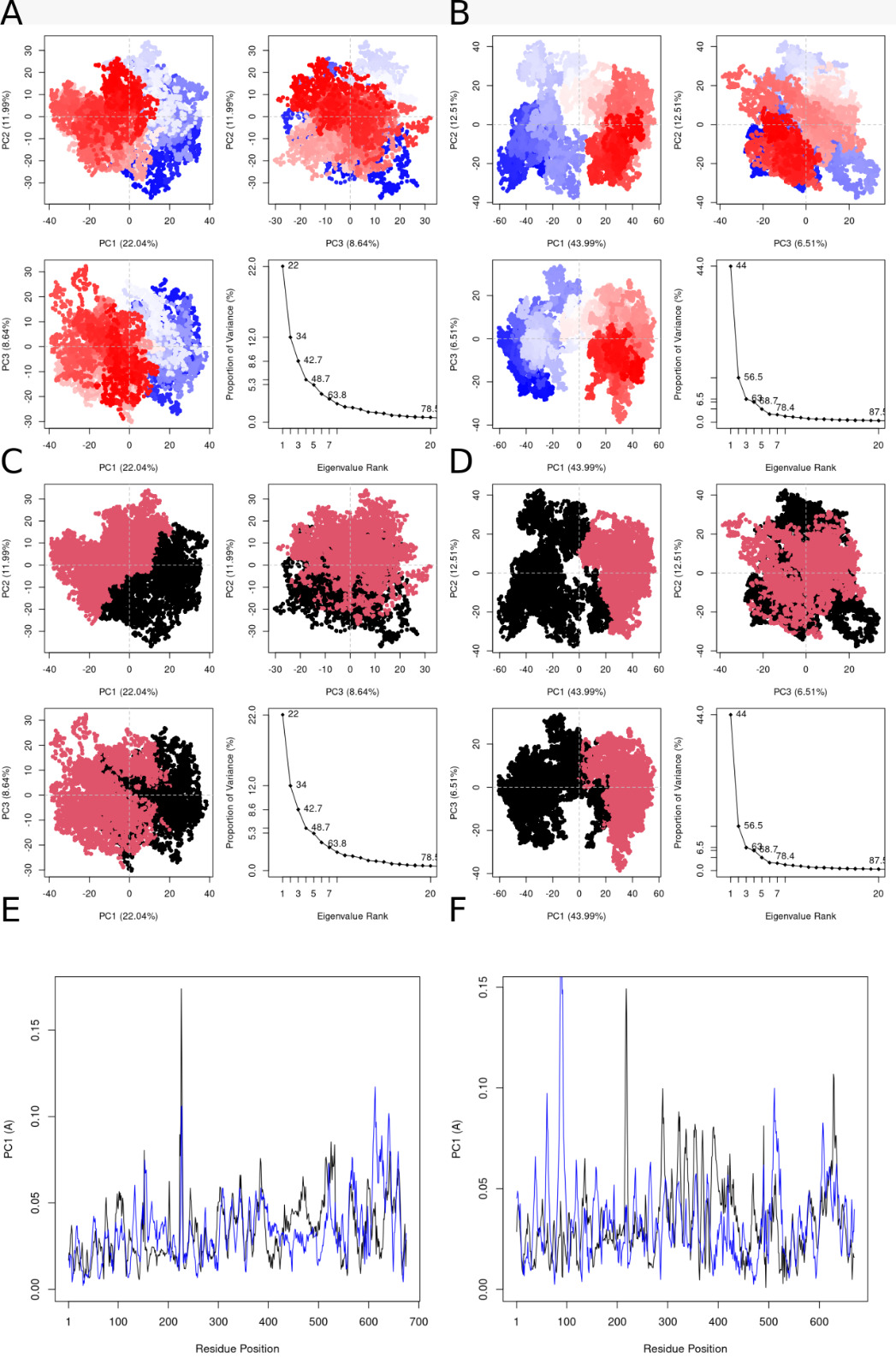

Supplement: Supplementary file 8 — Additional file 8. Supplementary figure 8: Bio3D analyses 2. A: DENV PCA (PC1, PC2 and PC3 correlations) and Eigenvalues for the 100 ns of molecular dynamics simulation. B: ZIKV PCA (PC1, PC2 and PC3 correlations) and Eigenvalues for the 100 ns of molecular dynamics simulation. C: DENV Hierarchical clustering (PC1, PC2 and PC3correlations) and Eigenvalues for the 100 ns of molecular dynamics simulation. D: ZIKV Hierarchical clustering (PC1, PC2 and PC3 correlations) and Eigenvalues for the 100 ns of molecular dynamics simulation. E: DENV -PC1 applied for residue position. F: ZIKV - PC1 applied for residue position. [file 12985_2023_2251_MOESM8_ESM.jpeg]

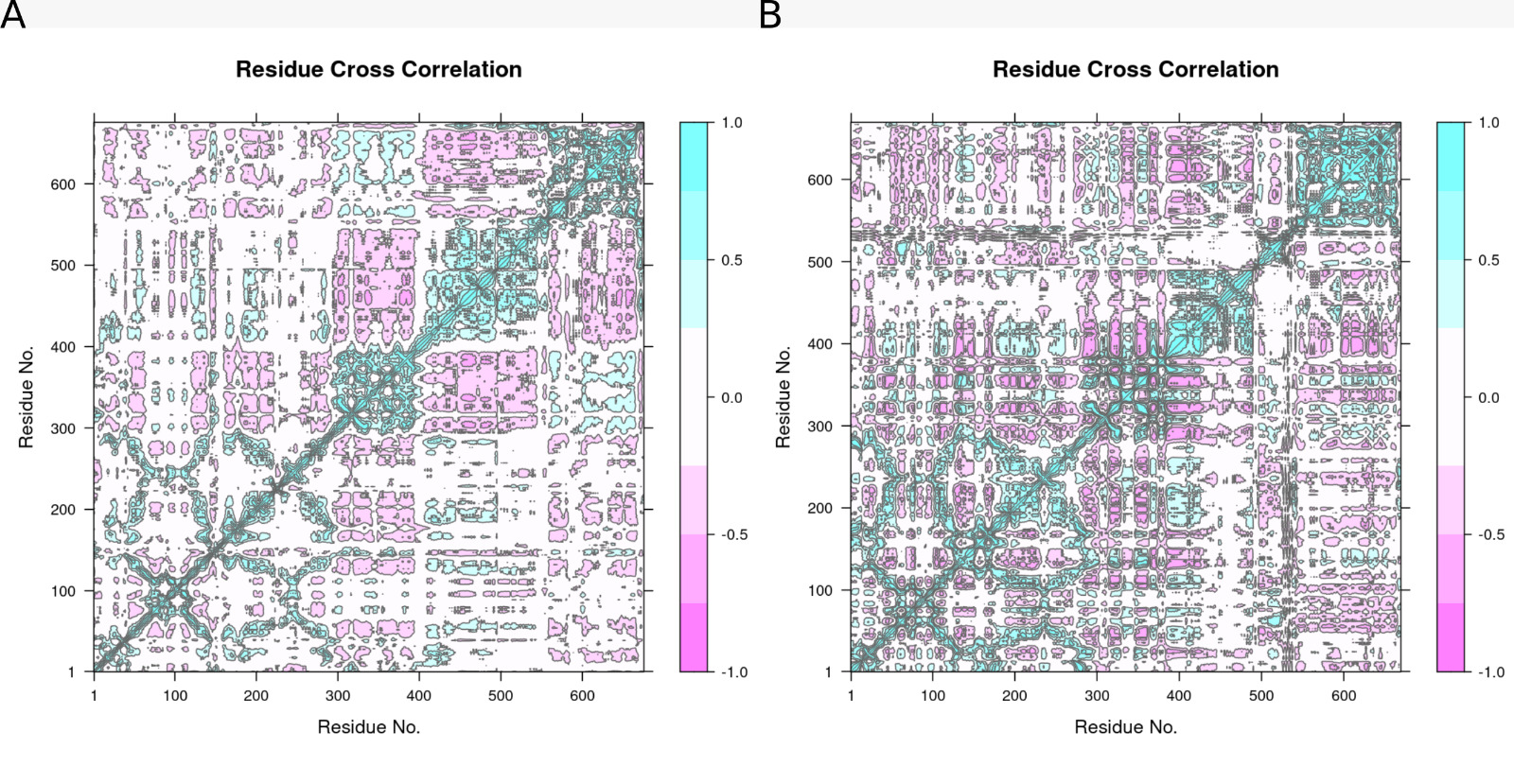

Supplement: Supplementary file 9 — Additional file 9. Supplementary figure 9: Residue cross correlation. A: DENV. B: ZIKV. [file 12985_2023_2251_MOESM9_ESM.jpeg]
